# Hypertension moderates the relationship between plasma beta-amyloid and cognitive impairment: a cross-sectional study in Xi’an, China

**DOI:** 10.3389/fnagi.2025.1532676

**Published:** 2025-01-28

**Authors:** Ziyu Liu, Yaoli He, Simeng Cui, Liangjun Dang, Binyan Zhang, Jin Wang, Wenhui Lu, Kang Huo, Yu Jiang, Chen Chen, Ling Gao, Shan Wei, Yi Zhao, Ningwei Hu, Jingyi Wang, Hong Lv, Qiumin Qu, Suhang Shang

**Affiliations:** ^1^Department of Neurology, The First Affiliated Hospital of Xi’an Jiaotong University, Xi’an, China; ^2^Department of Neurology, Baoji Central Hospital, Baoji, China; ^3^Department of Epidemiology and Health Statistics, School of Public Health, Xi’an Jiaotong University Health Science Center, Xi’an, China; ^4^Department of Neurology, The Second Affiliated Hospital of Xi’an Jiaotong University, Xi’an, China; ^5^Department of Neurology, Huyi Hospital of Traditional Chinese Medicine, Xi’an, China; ^6^Department of Neurology, The First Affiliated Hospital of China Medical University, Shenyang, China; ^7^Development and Related Diseases of Women and Children Key Laboratory of Sichuan Province, Chengdu, Sichuan, China; ^8^Center for Brain Science, The First Affiliated Hospital of Xi’an Jiaotong University, Xi’an, China

**Keywords:** beta-amyloid, cognitive impairment, hypertension, Alzheimer’s disease, a cross-sectional study

## Abstract

**Background:**

Plasma beta-amyloid (Aβ) are important biomarkers for Alzheimer’s disease and cognitive impairment (CI), but results are controversial. It remains unclear whether hypertension modulates their relationship. This cross-sectional study investigates whether hypertension moderates the relationship between plasma Aβ and cognitive impairment (CI).

**Methods:**

This cross-sectional study included 1488 subjects ≥ 40 years from rural areas of northwestern China. CI was defined as a Mini-Mental State Examination score lower than the cutoff. Firstly, plasma Aβ_40_, Aβ_42_, Aβ_42_/Aβ_40_ were analyzed as restricted cubic spline. Then, categories of combined plasma Aβ were created by making bisection of plasma Aβ according to average and combining them as L-Aβ_40_ and L-Aβ_42_, H-Aβ_40_ and L-Aβ_42_, L-Aβ_40_ and H-Aβ_42_, H-Aβ_40_ and H-Aβ_42_. Decreased plasma Aβ_40_ was defined as < 25th percentile. Multivariate logistic regression examined the relationship between plasma Aβ and CI in total population, the hypertension subgroup and the non-hypertension subgroup.

**Results:**

737 participants (49.5%) had hypertension and 189 participants (12.7%) had CI. Simultaneously elevated plasma Aβ_40_ and Aβ_42_ was associated with CI in hypertension (H-Aβ_40_ and H-Aβ_42_ vs. L-Aβ_40_ and L-Aβ_42_, 21.1% vs.10.7%, *P* = 0.033; OR = 1.984 [95% CI, 1.067–3.691], *P* = 0.030) but not in the non-hypertension. Decreased plasma Aβ_40_ was associated with CI in the non-hypertension (14.9% vs. 9.2%, *P* = 0.026; OR = 1.728 [95% CI, 1.018–2.931], *P* = 0.043) but not in the hypertension.

**Conclusion:**

Hypertension is an important modulator in the relationship between plasma Aβ and CI. Simultaneously elevated plasma Aβ_40_ and Aβ_42_ in the hypertension, and decreased plasma Aβ_40_ in the non-hypertension, may be risk factors for CI. These findings emphasize the need to consider hypertension in CI detection.

## 1 Introduction

Alzheimer’s disease (AD) is a serious condition that poses significant risks to the health and life of older adults, with effective treatment options remaining limited. Therefore, early diagnosis and delay of progression have become key prevention strategies (Livingston et al., 2020). Currently, central nervous system markers, such as beta-amyloid (Aβ) deposition detected by Positron Emission Tomography-Computed Tomography (PET-CT), or Aβ_42_ and Aβ_42_/Aβ_40_ levels in cerebrospinal fluid, are considered relatively reliable biomarkers for the early stage of AD (Jack et al., 2018). However, their widespread use is constrained by the invasive nature of procedures and the high equipment requirements. Therefore, researchers have focused on identifying peripheral biomarkers for AD, given the easier accessibility of test sample. Recent studies have some promising findings, with markers like plasma P-tau 181 and plasma P-tau 217 showing good diagnostic efficacy for AD (Janelidze et al., 2020; Palmqvist et al., 2020).

Aβ deposition is a key pathological feature of AD, making plasma Aβ a potential peripheral biomarker of interest (Sullivan et al., 2021). Recent studies have suggested that plasma Aβ levels correlate with AD-related central nervous system biomarkers, such as brain Aβ deposition (Botella Lucena et al., 2022), cerebrospinal fluid biomarkers (Aβ_42_, total Tau, P-tau) (Hanon et al., 2018; Teunissen et al., 2018), and hippocampal volume (Hilal et al., 2018), supporting the possibility that plasma Aβ may be associated with AD or cognitive dysfunction. However, results from population-based studies on the relationship between plasma Aβ levels and AD or cognitive impairment have been inconsistent (Brickman et al., 2021; Chen et al., 2019; Cullen et al., 2021; de Wolf et al., 2020; Giudici et al., 2020; Rembach et al., 2014). These previous studies indicate that the relationship between plasma Aβ and AD or cognitive function is complex, involving more than a straightforward positive or negative correlation (Botella Lucena et al., 2022; Schupf et al., 2008).

Many studies, including population and animal studies, have confirmed that hypertension is one of the risk factors for AD (Livingston et al., 2020; Scheltens et al., 2021). Some studies also find that hypertension could contribute to elevated plasma Aβ levels (Abdullah et al., 2009; Lambert et al., 2011), raising the question: does hypertension alter the relationship between plasma Aβ levels and cognitive impairment? In rural areas, specific healthcare challenges and demographic characteristics may influence the interplay between hypertension and cognitive impairment. In light of this, our study aimed to analyze the relationship between plasma Aβ and cognitive impairment in a general population in rural areas of Northwestern China, and a stratified analysis was performed to assess whether the associations were affected by the hypertension.

## 2 Materials and methods

### 2.1 Data sources and study population

The data were obtained from a cross-sectional, cluster sampling study on cerebrovascular disease and cognitive impairment, which was conducted at a village in the suburbs of Xi’an, northwestern China between October 8, 2014 and March 30, 2015. Detailed protocol has been described previously (Shang et al., 2016).

**FIGURE 1:**
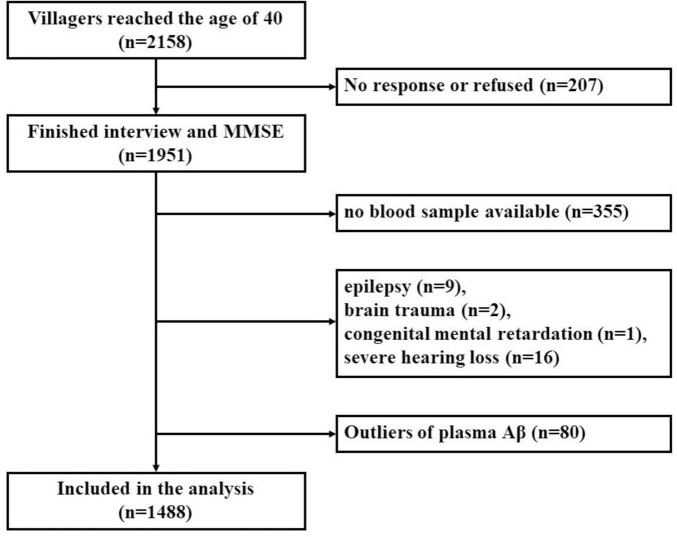
Flow chart of participants selection.

The inclusion criteria for the present study were as follows: (1) permanent residents of the selected village; (2) subjects ≥ 40 years old. The exclusion criteria were as follows: (1) subjects who had no response, or refused to participate in this research; (2) individuals who were suffering from other medical conditions that may affect cognitive function, such as chronic alcoholism, brain trauma, past craniocerebral operations, central nervous system tumor, intracranial infection, epilepsy (all types), organic psychosis, schizophrenia, affective psychosis, congenital intellectual disability, or untreated hypothyroidism; (3) subjects with severe visual or hearing dysfunction that may preclude cognitive testing; (4) subjects who were suffering from severe cardiac disease, hepatic disease, renal disease, pulmonary disease, hematological disease or acute or end-stage of various chronic diseases; (5) subjects who refused to take blood samples; (6) subjects whose plasma Aβ data were missing, or plasma Aβ were defined as outliers (exceeding ± 3 standard deviations (SDs) from the mean). The detailed screening process of the participants is shown in Figure 1. This study was approved by the Medical Ethics Committee of the First Affiliated Hospital of Xi’an Jiaotong University. Written informed consent of all participants was obtained.

### 2.2 Definition of hypertension

Hypertension was defined as either twice elevated blood pressure (systolic blood pressure (SBP) ≥ 140 mm Hg or diastolic blood pressure (DBP) ≥ 90 mm Hg) measured on different days or sure history of hypertension reported by participants or the caregivers (James et al., 2014).

BP was measured 2 times in seated position using a mercury sphygmomanometer, after the participant had rested for 10 minutes and refrained from vigorous exercise for at least 30 minutes prior to each measurement. If the mean of two BP measurements was elevated (systolic BP ≥ 140 mmHg or diastolic BP ≥ 90 mm Hg), the BP measurement procedure was repeated on a separate day. This criterion was selected to minimize measurement variability and better reflect chronic hypertension.

### 2.3 Plasma Aβ detection and classification

All measurements were conducted using standard instruments and adhered to strict protocols in both preanalytical processes and measurement procedures (Watt et al., 2012). Details on the measurement of plasma Aβ have been described previously (Jiang et al., 2018). The levels of plasma Aβ_40_ and plasma Aβ_42_ were measured using commercially available quantitative enzyme-linked immunosorbent assay kits (ELISA, Yuanye Co., Shanghai, China). Measurements were performed using an RT-6000 analyzer (Rayto Co., Shenzhen, China) at 450 nm, and concentrations were calculated from the standard curve. All measurements were performed in duplicate, and the results averaged. The intra-assay and inter-assay coefficients of variation were less than 7 and 9%, respectively.

Since there is currently no established normal cut-off value for plasma Aβ levels, plasma Aβ classifications in this study were based on the preliminary analyses results (See in Statistics analysis and Results). Plasma Aβ categories were created by bisecting plasma Aβ_40_ and plasma Aβ_42_ values around their respective averages: low plasma Aβ_40_ (L-Aβ_40_) was defined as < 52 pg/ml and high plasma Aβ_40_ (H-Aβ_40_) as ≥ 52 pg/ml; low plasma Aβ_42_ (L-Aβ_42_) was defined as < 41 pg/ml and high plasma Aβ_42_ (H-Aβ_42_) as ≥ 41 pg/ml. These levels were then combined into categories: L-Aβ_40_ and L-Aβ_42_, H-Aβ_40_ and L-Aβ_42_, L-Aβ_40_ and H-Aβ_42_, and H-Aβ_40_ and H-Aβ_42_. Additionally, decreased plasma Aβ_40_ was defined as plasma Aβ_40_ lower than 25th percentile (46 pg/ml). The 25th percentile was selected based on prior research indicating that the lowest Aβ40 tertile could predict incident AD (Sundelöf et al., 2008).

### 2.4 Cognitive assessment

Chinese version of the Mini-Mental State Examination (MMSE) was used to assess the global cognitive function (Katzman et al., 1988), addressing language barriers and cultural differences in this population. To enhance the sensitivity and specificity of the assessment, cognitive impairment was defined using cutoff values adjusted for differences in education levels: scores ≤ 17 for subjects who were illiterate, scores ≤ 20 for subjects with a primary school education, and scores ≤ 24 for subjects with a junior high school education or above (Katzman et al., 1988), same with our previous studies (Shang et al., 2016).

### 2.5 Covariates

Covariates included demographic information (sex, age, years of education), health-related lifestyle (tobacco use, alcohol consumption, physical exercise), comorbidities (hypertension, diabetes, coronary heart disease, dyslipidemia, transient ischemic attack and stroke), family history (hypertension, diabetes, coronary heart disease and stroke) and biochemical indicators (fasting blood glucose (FBG), total cholesterol (TC), triglyceride (TG), high-density lipoprotein cholesterol (HDL), and low-density lipoprotein cholesterol (LDL) levels).

### 2.6 Statistical analysis

Statistical analyses were performed with SPSS 18.0 statistical software and R software (version 4.0.3). GraphPad Prism 8 and R software were used for graphing. The characteristics were reported as the mean ± SDs for approximately normally distributed data, the median (25th percentile, 75th percentile) for severely skewed data, and numerical values (percentages) for categorical data. In the univariate analysis, differences were evaluated using t tests, one-way ANOVA, χ^2^-tests, and rank tests according to data type and distribution. Multivariate logistic regression models were established with cognitive impairment (yes or no) as the dependent variable, with plasma Aβ indicators as the independent variables, and with sex, age, years of education, smoking, drinking, lack of physical activity, heart disease, stroke, mean arterial pressure, body mass index (BMI), FBG, TG, TC, LDL, and HDL as covariates to calculate Odds Ratio (OR) and 95% confidence interval (CI). All statistical tests were two-tailed, and statistical significance was set at 5%.

The analyses steps were as follows. First, the preliminary analyses were performed using multivariate logistic regression models with plasma Aβ_40_, Aβ_42_ or Aβ_42_/Aβ_40_ fitted as restricted cubic splines, respectively, to explore the risk of cognitive impairment under different plasma Aβ levels, as well as the potential non-linear associations. Next, four combined categories of plasma Aβ (L-Aβ_40_ and L-Aβ_42_, H-Aβ_40_ and L-Aβ_42_, L-Aβ_40_ and H-Aβ_42_, H-Aβ_40_ and H-Aβ_42_) was established by categorizing Aβ_40_ and Aβ_42_ as binary variables based on their average values. The prevalence of cognitive impairment among the four groups was then compared. Multivariate logistic regression models were established to adjust for confounding factors, with the four plasma Aβ categories included as a dummy variable, using the L-Aβ_40_ and L-Aβ_42_ group as the reference category. Finally, decreased plasma Aβ_40_ was defined as levels lower than 25th percentile (46 pg/ml) based on preliminary analyses and published research, and the relationship between decreased plasma Aβ_40_ and cognitive impairment was analyzed. All analyses were firstly performed in the total population, followed by stratified analyses based on hypertension status.

## 3 Results

### 3.1 Demographic and clinical information

A total of 1488 subjects aged 40-85 (55.81 ± 10.06) years, were analyzed in this study. There were 590 males (39.7%) and 737 hypertensive individuals (49.5%). Plasma Aβ_40_ (52.49 ± 8.99 pg/ml), plasma Aβ_42_ (40.95 ± 6.72 pg/ml) and plasma Aβ_42_/Aβ_40_ (0.80 ± 0.20) were normally distributed. MMSE score was skewed distributed [Median (P25, P75), 27 (24,29)], and 189 participants (12.7%) were diagnosed with cognitive impairment according to the criteria described above. The demographics and clinical characteristics of the participants are presented in Table 1. The comparison of normal cognition group and cognitive impairment group are presented in Table 2.

**TABLE 1:** Demographic and clinical characteristics of the study population.

Variables	Total (*n* = 1488)	Non-HP (*n* = 751)	HP (*n* = 737)	*P*
Male [n(%)]	590(39.7)	302(40.2)	288(39.1)	0.645
Age [Mean(SD), year]	55.81(10.06)	52.79(9.43)	58.89(9.75)	< 0.001
Formal education [n(%)]				< 0.001
Uneducated	199(13.4)	65(8.7)	134(18.2)	
Primary school	428(28.8)	197(26.2)	231(31.3)	
High school or above	861(57.9)	489(65.1)	372(50.5)	
Years of education [Median(P25,P75), year]	7(4,8)	8(5,9)	6(3,8)	< 0.001
Marital status [n(%)]				0.003
Married	1375(92.4)	709(94.4)	666(90.4)	
Others	113(7.6)	42(5.6)	71(9.6)	
Tobacco use [n(%)]	422(28.4)	219(29.2)	203(27.5)	0.489
Alcohol consumption [n(%)]	202(13.6)	106(14.1)	96(13.0)	0.540
Lack of physical activity [n(%)]	266(17.9)	106(14.1)	160(21.7)	< 0.001
**Comorbidities [n(%)]**				
HP	737(49.5)	–	–	–
DM	186(12.5)	52(6.9)	134(18.2)	< 0.001
Dyslipidemia	768(51.6)	321(42.7)	447(60.7)	< 0.001
HD	98(6.6)	31(4.1)	67(9.1)	< 0.001
Atrial fibrillation	12(0.8)	2(0.3)	10(1.4)	0019
TIA	28(1.9)	8(1.1)	20(2.7)	0.019
Stroke	106(7.1)	24(3.2)	82(11.1)	< 0.001
Antihypertensive drugs [n(%)]	240(16.1)	0(0)	240(32.6)	< 0.001
Hypoglycemic drugs [n(%)]	83(5.6)	21(2.8)	62(8.4)	< 0.001
Antiplatelet drugs [n(%)]	69(4.6)	11(1.5)	58(7.9)	< 0.001
Statins [n(%)]	54(3.6)	10(1.3)	44(6.0)	< 0.001
SBP [Mean(SD), mmHg]	132.66(18.75)	119.35(9.86)	146.22(15.69)	< 0.001
DBP [Mean(SD), mmHg]	82.20(10.32)	75.97(6.30)	88.53(9.75)	< 0.001
BMI[Mean(SD), kg/m^2^]	25.30(3.21)	24.54(2.88)	26.08(3.34)	< 0.001
**Biochemical examination**				
FBG [Median(P25,P75),mmol/L]	5.4(5.07,5.81)	5.30(4.99,5.62)	5.51(5.16,6.07)	< 0.001
TG [Median(P25,P75),mmol/L]	1.44(1.03,2.01)	1.26(0.95,1.75)	1.63(1.19,2.21)	< 0.001
TC [Mean(SD), mmol/L]	5.02(1.01)	4.92(0.97)	5.12(1.04)	< 0.001
LDL [Mean(SD), mmol/L]	3.29(0.90)	3.21(0.88)	3.37(0.91)	0.001
HDL [Mean(SD), mmol/L]	1.41(0.31)	1.42(0.31)	1.39(0.31)	0.089
MMSE score [Median(P25,P75)]	27(24,29)	27(25,29)	26(23,28)	< 0.001
Cognitive impairment [n(%)]	189(12.7)	80(10.7)	109(14.8)	0.017
plasma Aβ_40_ [Mean(SD), pg/ml]	52.49(8.99)	52.10(9.11)	52.89(8.86)	0.091
plasma Aβ_42_ [Mean(SD), pg/ml]	40.95(6.72)	41.00(6.67)	40.91(6.77)	0.797
plasma Aβ_42_/Aβ_40_ [Mean(SD)]	0.80(0.20)	0.81(0.19)	0.80(0.20)	0.178
Categories of combined plasma Aβ [n(%)]				0.145
L-Aβ_40_ and L-Aβ_42_	377(25.3)	208(27.7)	169(22.9)	
H-Aβ_40_ and L-Aβ_42_	382(25.7)	181(24.1)	201(27.3)	
L-Aβ_40_ and H-Aβ_42_	343(23.1)	166(22.1)	177(24.0)	
H-Aβ_40_ and H-Aβ_42_	386(25.9)	196(26.1)	190(25.8)	

Non-HP, non-hypertension. HP, hypertension. DM, diabetes. HD, heart disease. TIA, transient ischemic attack. SBP, systolic blood pressure. DBP, diastolic blood pressure. FBG, fasting blood glucose. TC, total cholesterol. TG, triglycerides. LDL, low-density lipoprotein cholesterol. HDL, high-density lipoprotein cholesterol. MMSE, Mini-Mental State Examination. Categories of combined plasma Aβ were created by making bisection of plasma Aβ_40_ (L-Aβ_40_ < 52 pg/ml and H-Aβ_40_ ≥ 52 pg/ml) and plasma Aβ_42_ (L-Aβ_42_ < 41 pg/ml and H-Aβ_42_ ≥ 41 pg/ml) according to average value, and combining them as L-Aβ_40_ and L-Aβ_42_, H-Aβ_40_ and L-Aβ_42_, L-Aβ_40_ and H-Aβ_42_, H-Aβ_40_ and H-Aβ_42_.

**TABLE 2:** Comparison of the normal cognition group and the cognitive impairment group.

Variables	Total (*n* = 1,488)	Normal cognition (*n* = 1,299)	Cognitive impairment (*n* = 189)	*P*
Male [n(%)]	590(39.7)	512(39.4)	78(41.3)	0.626
Age [Mean(SD), year]	55.81(10.06)	54.89(9.54)	62.19(11.23)	< 0.001
Formal education [n(%)]				< 0.001
Uneducated	199(13.4)	143(11.0)	56(29.6)	
Primary school	428(28.8)	372(28.6)	56(29.6)	
High school or above	861(57.9)	784(60.4)	77(40.7)	
Years of education [Median(P25,P75), year]	7(4,8)	7(5,9)	5(0,8)	< 0.001
Marital Status [n(%)]				< 0.001
Married	1375(92.4)	1215(93.5)	160(84.7)	
Other	113(7.6)	84(6.5)	29(15.3)	
Tobacco use [n(%)]	422(28.4)	365(28.1)	57(30.2)	0.557
Alcohol consumption [n(%)]	202(13.6)	178(13.7)	24(12.7)	0.706
Lack of physical activity [n(%)]	266(17.9)	226(17.4)	40(21.2)	0.207
**Comorbidities [n(%)]**				
HP	737(49.5)	628(48.3)	109(57.7)	0.017
DM	186(12.5)	151(11.6)	35(18.5)	0.007
Dyslipidemia	768(51.6)	664(51.1)	104(55.0)	0.315
HD	98(6.6)	78(6.0)	20(10.6)	0.018
Atrial fibrillation	12(0.8)	9(0.7)	3(1.6)	0.188
TIA	28(1.9)	25(1.9)	3(1.6)	1.000
Stroke	106(7.1)	85(6.5)	21(11.1)	0.023
Antihypertensive drugs [n(%)]	240(16.1)	202(15.6)	38(20.1)	0.112
Hypoglycemic drugs [n(%)]	83(5.6)	70(5.4)	13(6.9)	0.404
Antiplatelet drugs [n(%)]	69(4.6)	57(4.4)	12(6.3)	0.231
Statins [n(%)]	54(3.6)	45(3.5)	9(4.8)	0.373
SBP [Mean(SD), mmHg]	132.66(18.75)	132.02(18.60)	137.04(19.19)	0.001
DBP [Mean(SD), mmHg]	82.20(10.32)	82.00(10.20)	83.54(11.05)	0.055
BMI[Mean(SD), kg/m^2^]	25.30(3.21)	25.39(3.18)	24.69(3.36)	0.005
**Biochemical examination**				
FBG [Median(P25,P75),mmol/L]	5.4(5.07,5.81)	5.39(5.07,5.80)	5.42(5.07,6.02)	0.387
TG [Median(P25,P75),mmol/L]	1.44(1.03,2.01)	1.44(1.04,2.00)	1.44(1.00,2.05)	0.898
TC [Mean(SD), mmol/L]	5.02(1.01)	5.01(1.00)	5.09(1.11)	0.371
LDL [Mean(SD), mmol/L]	3.29(0.90)	3.29(0.88)	3.31(0.99)	0.791
HDL [Mean(SD), mmol/L]	1.41(0.31)	1.40(0.31)	1.44(0.33)	0.087
MMSE score [Median(P25,P75)]	27(24,29)	27(25,29)	18(15,23)	< 0.001
Cognitive impairment [n(%)]	189(12.7)	–	–	–
plasma Aβ_40_ [Mean(SD), pg/ml]	52.49(8.99)	52.49(8.87)	52.45(9.77)	0.949
plasma Aβ_42_ [Mean(SD), pg/ml]	40.95(6.72)	40.84(6.65)	41.75(7.13)	0.081
plasma Aβ_42_/Aβ_40_ [Mean(SD)]	0.80(0.20)	0.80(0.19)	0.83(0.22)	0.107
Categories of combined plasma Aβ [n(%)]				0.250
L-Aβ_40_ and L-Aβ_42_	377(25.3)	334(25.7)	43(22.8)	
H-Aβ_40_ and L-Aβ_42_	382(25.7)	339(26.1)	43(22.8)	
L-Aβ_40_ and H-Aβ_42_	343(23.1)	300(23.1)	43(22.8)	
H-Aβ_40_ and H-Aβ_42_	386(25.9)	326(25.1)	60(31.7)	

Non-HP, non-hypertension. HP, hypertension. DM, diabetes. HD, heart disease. TIA, transient ischemic attack. SBP, systolic blood pressure. DBP, diastolic blood pressure. FBG, fasting blood glucose. TC, total cholesterol. TG, triglycerides. LDL, low-density lipoprotein cholesterol. HDL, high-density lipoprotein cholesterol. MMSE, Mini-Mental State Examination. Categories of combined plasma Aβ were created by making bisection of plasma Aβ_40_ (L-Aβ_40_ < 52 pg/ml and H-Aβ_40_ ≥ 52 pg/ml) and plasma Aβ_42_ (L-Aβ_42_ < 41 pg/ml and H-Aβ_42_ ≥ 41 pg/ml) according to average value, and combining them as L-Aβ_40_ and L-Aβ_42_, H-Aβ_40_ and L-Aβ_42_, L-Aβ_40_ and H-Aβ_42_, H-Aβ_40_ and H-Aβ_42_.

### 3.2 Preliminary analyses of the associations between plasma Aβ and cognitive impairment

**FIGURE 2:**
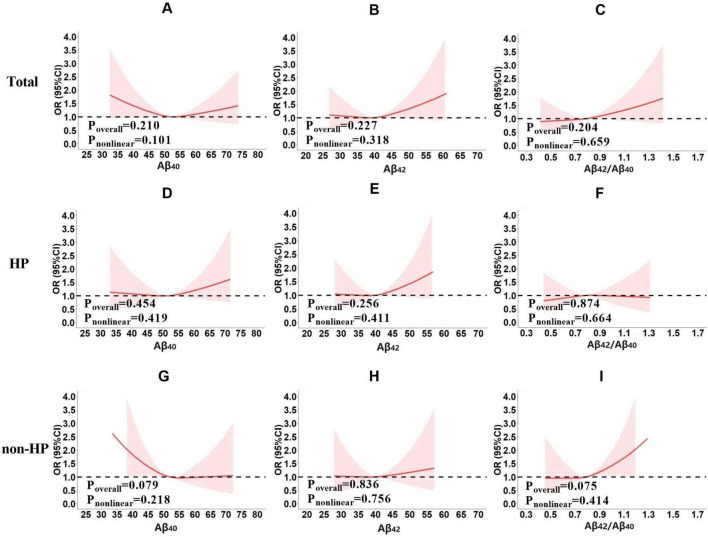
The risk of cognitive impairment under different plasma Aβ (Aβ_40_, Aβ_42_ or Aβ_42_/Aβ_40_) levels in total population **(A–C)**, the hypertension subgroup **(D–F)** and non-hypertension subgroup **(G–I)**. The preliminary analyses were performed using multivariate logistic regression models with cognitive impairment (yes or no) as the dependent variable, plasma Aβ_40_, Aβ_42_ or Aβ_42_/Aβ_40_ fitted as restricted cubic splines, respectively. Sex, age, years of education, smoking, drinking, lack of physical activity, heart disease, stroke, mean arterial pressure, BMI, FBG, TG, TC, LDL, and HDL were corrected.

In the preliminary analyses, multivariate logistic regression models were established with cognitive impairment (yes or no) as the dependent variable, plasma Aβ (Aβ_40_, Aβ_42_ and Aβ_42_/Aβ_40_, respectively) as the independent variable (fitted as restricted cubic splines), and other potential confounding factors (age, sex, years of education, smoking, drinking, lack of physical activity, heart disease, stroke, mean arterial pressure, BMI, FBG, TG, TC, LDL, and HDL) as covariates (Figure 2). These analyses were performed in the total population first and then in subgroups based on the hypertension status.

Results showed that no significant correlation was found between plasma Aβ and cognitive impairment in the total population (Aβ_40_, P_overall_ = 0.210, P_nonlinear_ = 0.101, Figure 2A; Aβ_42_, P_overall_ = 0.227, P_nonlinear_ = 0.318, Figure 2B; Aβ_42_/Aβ_40_, P_overall_ = 0.204, P_nonlinear_ = 0.659, Figure 2C), hypertensive subgroup (Aβ_40_, P_overall_ = 0.454, P_nonlinear_ = 0.419, Figure 2D; Aβ_42_, P_overall_ = 0.256, P_nonlinear_ = 0.411, Figure 2E; Aβ_42_/Aβ_40_, P_overall_ = 0.874, P_nonlinear_ = 0.664, Figure 2F), or non-hypertensive subgroup (Aβ_40_, P_overall_ = 0.079, P_nonlinear_ = 0.218, Figure 2G; Aβ_42_, P_overall_ = 0.836, P_nonlinear_ = 0.756, Figure 2H; Aβ_42_/Aβ_40_, P_overall_ = 0.075, P_nonlinear_ = 0.414, Figure 2I).

Preliminary analyses did not identify a significant association between plasma Aβ and cognitive impairment, but suggested some intriguing trends. It appeared that the relationship between cognitive impairment risk and plasma Aβ levels may be influenced by hypertension. In the hypertensive subgroup, there was a tendency for the risk of cognitive impairment to increase with rising plasma Aβ_40_ (Figure 2D) and Aβ_42_ levels (Figure 2E). In the non-hypertensive subgroup, however, the risk of cognitive impairment appeared higher at lower plasma Aβ_40_ levels, decreased as plasma Aβ_40_ levels rose to the mean, and then remained stable (Figure 2G). Although these associations were not statistically significant, the observed trends suggested a potential biological trend worth further exploration.

### 3.3 Simultaneously elevated plasma Aβ_40_ and Aβ_42_ was associated with cognitive impairment in hypertension but not in non-hypertension

**FIGURE 3:**
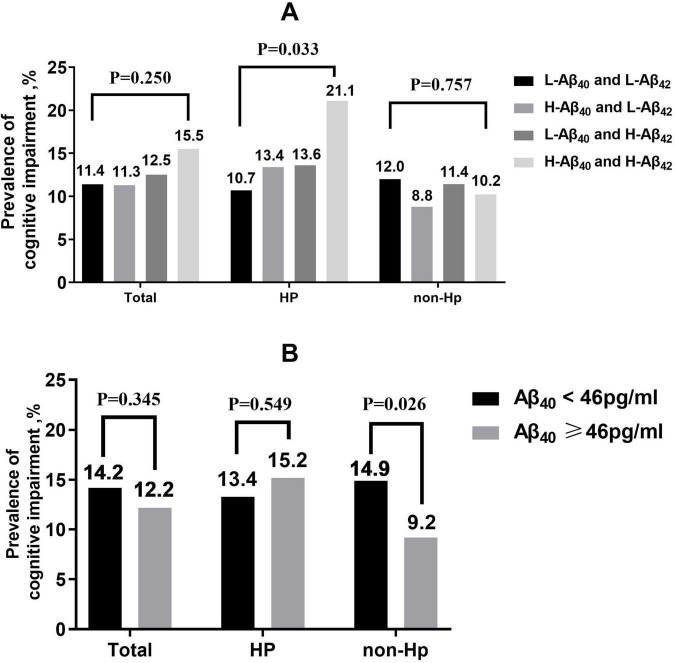
The prevalence of cognitive impairment according to four categories of combined plasma Aβ **(A)** and decreased plasma Aβ_40_ **(B)** in total population, hypertension subgroup and non-hypertensive subgroup. Categories of combined plasma Aβ were created by making bisection of plasma Aβ_40_ (L-Aβ_40_ < 52 pg/ml and H-Aβ_40_ ≥ 52 pg/ml) and plasma Aβ_42_ (L-Aβ_42_ < 41 pg/ml and H-Aβ_42_ ≥ 41 pg/ml) according to average value, and combining them as L-Aβ_40_ and L-Aβ_42_, H-Aβ_40_ and L-Aβ_42_, L-Aβ_40_ and H-Aβ_42_, H-Aβ_40_ and H-Aβ_42_. Decreased plasma Aβ40 was defined as < 25th percentile (46 pg/ml).

Based on the results above, we further analyzed whether the simultaneous increase in plasma Aβ_40_ and Aβ_42_ levels was associated with cognitive impairment. Four combined plasma Aβ categories were created by bisecting plasma Aβ_40_ into low (L-Aβ_40_ < 52 pg/ml) and high (H-Aβ_40_ ≥ 52 pg/ml) levels, and plasma Aβ_42_ into low (L-Aβ_42_ < 41 pg/ml) and high (H-Aβ_42_ ≥ 41 pg/ml) levels, based on their average values. These were then combined into four groups: L-Aβ_40_ and L-Aβ_42_, H-Aβ_40_ and L-Aβ_42_, L-Aβ_40_ and H-Aβ_42_, and H-Aβ_40_ and H-Aβ_42_. There were significant differences in the prevalence of cognitive impairment among the four groups in the hypertension subgroup (L-Aβ_40_ and L-Aβ_42_ vs. H-Aβ_40_ and L-Aβ_42_ vs. L-Aβ_40_ and H-Aβ_42_ vs. H-Aβ_40_ and H-Aβ_42_, 10.7% vs. 13.4% vs. 13.6% vs. 21.1%, *P* = 0.033, Figure 3A). After adjusting for confounding factors, the risk of cognitive impairment in the H-Aβ_40_ and H-Aβ_42_ group was significantly higher than that in the L-Aβ_40_ and L-Aβ_42_ group (OR = 1.984 [95% CI, 1.067–3.691], *P* = 0.030, Table 3). However, no similar association was found in the non-hypertensive subgroup (Figure 3A; Table 3). This lack of association may indicate differences in Aβ clearance or metabolic mechanisms between hypertensive and non-hypertensive individuals.

**TABLE 3:** Risk of cognitive impairment at different levels of plasma Aβ in total population, hypertension subgroup and non-hypertension subgroup.

Variables	B	S.E.	Wald	OR	95% CI	*P*
**Categories of combined plasma Aβ in total**						
L-Aβ_40_ and L- Aβ_42_ (reference)	0	–	–	1	–	–
H-Aβ_40_ and L- Aβ_42_	-0.129	0.240	0.289	0.879	0.549–1.407	0.591
L-Aβ_40_ and H- Aβ_42_	-0.008	0.241	0.001	0.992	0.619–1.590	0.973
H-Aβ_40_ and H- Aβ_42_	0.264	0.225	1.372	1.302	0.837–2.024	0.242
**Categories of combined plasma Aβ in HP**						
L-Aβ_40_ and L- Aβ_42_ (reference)	0	–	–	1	–	–
H-Aβ_40_ and L- Aβ_42_	0.162	0.338	0.231	1.176	0.607–2.280	0.630
L-Aβ_40_ and H- Aβ_42_	0.169	0.343	0.241	1.184	0.604–2.320	0.624
H-Aβ_40_ and H- Aβ_42_	0.685	0.317	4.683	1.984	1.067–3.691	0.030
**Categories of combined plasma Aβ in non-HP**						
L-Aβ_40_ and L- Aβ_42_ (reference)	0	–	–	1	–	–
H-Aβ_40_ and L- Aβ_42_	-0.405	0.361	1.259	0.667	0.328–1.353	0.262
L-Aβ_40_ and H- Aβ_42_	-0.106	0.348	0.092	0.900	0.455–1.780	0.761
H-Aβ_40_ and H- Aβ_42_	-0.157	0.347	0.205	0.855	0.433–1.687	0.651
Decreased Aβ_40_ in total	0.201	0.187	1.152	1.223	0.847–1.766	0.283
Decreased Aβ_40_ in HP	-0.168	0.273	0.381	0.845	0.495–1.442	0.537
Decreased Aβ_40_ in non-HP	0.547	0.270	4.109	1.728	1.018–2.931	0.043

Multivariate logistic regression models were established with cognitive impairment (yes or no) as the dependent variable and plasma Aβ categories as the independent variable in total population, the hypertension subgroup and the non-hypertension subgroup. The adjusted confounders included age, sex, years of education, smoking, drinking, lack of physical activity, heart disease, stroke, mean arterial pressure, BMI, FBG, TG, TC, LDL, and HDL. Categories of combined plasma Aβ were created by making bisection of plasma Aβ_40_ (L-Aβ_40_ < 52 pg/ml and H-Aβ_40_ ≥ 52 pg/ml) and plasma Aβ_42_ (L-Aβ_42_ < 41 pg/ml and H-Aβ_42_ ≥ 41 pg/ml) according to average value, and combining them as L-Aβ_40_ and L-Aβ_42_, H-Aβ_40_ and L-Aβ_42_, L-Aβ_40_ and H-Aβ_42_, H-Aβ_40_ and H-Aβ_42_. Decreased plasma Aβ_40_ was defined as plasma Aβ_40_ lower than 25th percentile (<46 pg/ml).

In addition, we also created four combined plasma Aβ categories based on the 75th quantile (Aβ_40_, L- Aβ_40_ < 58 pg/ml vs. H-Aβ_40_ ≥ 58 pg/ml; Aβ_42_, L-Aβ_42_ < 45 pg/ml vs. H-Aβ_42_ ≥ 45 pg/ml) and repeated the above analyses, the results were similar (Supplementary Figure S1; Supplementary Table S1).

### 3.4 Decreased plasma Aβ_40_ was associated with increased risk of cognitive impairment in the non-hypertensive subgroup but not in the hypertensive subgroup

Inspired by the preliminary analyses, we also analyzed the relationship between decreased plasma Aβ_40_ and cognitive impairment in the total population, hypertensive subgroup, and non-hypertensive subgroup. Results showed that decreased plasma Aβ_40_ was significantly associated with an increased risk of cognitive impairment only in the non-hypertensive subgroup (Aβ_40_ < 46 pg/ml vs. Aβ_40_ ≥ 46 pg/ml, 14.9% vs. 9.2%, *P* = 0.026, Figure 3B) and not in the hypertensive subgroup (Aβ_40_ < 46 pg/ml vs. Aβ_40_ ≥ 46 pg/ml, 13.4% vs. 15.2%, *P* = 0.549, Figure 3B). After adjusting for confounding factors using multivariate logistic regression, a similar relationship remained in the non-hypertensive subgroup (OR = 1.728 [95% CI, 1.018–2.931], *P* = 0.043, Table 3) but not in the hypertensive subgroup (OR = 0.845 [95% CI, 0.495–1.442], *P* = 0.537, Table 3).

### 3.5 Sensitivity analyses

Sensitivity analyses were first conducted in participants without stroke history (left with 1382 participants). The results were similar with before: significant differences were found in the prevalence of cognitive impairment among the four groups in the hypertension subgroup (L-Aβ_40_ and L-Aβ_42_ vs. H-Aβ_40_ and L-Aβ_42_ vs. L-Aβ_40_ and H-Aβ_42_ vs. H-Aβ_40_ and H-Aβ_42_, 8.9% vs. 11.6% vs. 9.0% vs. 19.1%, *P* = 0.023); Multivariable logistic regressions showed that the risk of cognitive impairment in the H-Aβ_40_ and H-Aβ_42_ group was significantly higher than that in the L-Aβ_40_ and L-Aβ_42_ group (OR = 2.410 [95% CI, 1.125–5.163], *P* = 0.024, Supplementary Table S2); No significant association was found in the non-hypertensive subgroup (Supplementary Table S2). Interestingly, while the results for decreased plasma Aβ40 were no longer significant in the non-HP subgroup, in the HP subgroup, decreased plasma Aβ40 was significantly associated with a reduced risk of cognitive impairment (OR = 0.347 [95% CI, 0.157–0.767], *P* = 0.009, Supplementary Table S2). This finding suggested a complex interaction between different vascular factors and Aβ dynamics, which worth further exploration.

On the other hand, we also stratified the hypertensive individuals into treated-HP [240(32.6%)] and non-treated-HP groups to repeat the analyses. For categories of combined plasma Aβ, it showed that the significant associations observed previously remained marginally significant only in the non-treated-HP group (OR = 2.307 [95% CI, 0.991–5.374], *P* = 0.053, Supplementary Table S3) and not in the treated-HP group (OR = 2.607 [95% CI, 0.417–10.237], *P* = 0.374, Supplementary Table S3). This discrepancy could be partly due to the relatively small sample size in treated-HP group. However, a reasonable speculation could be that effective antihypertensive treatment may mitigate the detrimental effects of hypertension on Aβ dynamics and cognitive function, thereby weakening the observed associations. For decreased plasma Aβ_40_, the sample size was too small to do the analysis.

## 4 Discussion

This study found that the cross-sectional association between plasma Aβ levels and cognitive impairment was modulated by hypertension. In the hypertensive subgroup, individuals with simultaneously elevated plasma Aβ_40_ and Aβ_42_ levels exhibited a significantly higher risk of cognitive impairment; however, this association was not observed in the non-hypertensive subgroup. In contrast, decreased plasma Aβ_40_ was significantly associated with an increased risk of cognitive impairment in the non-hypertensive subgroup, while no such association was found in the hypertensive subgroup. No significant association between plasma Aβ_42_, the plasma Aβ_42_/Aβ_40_ ratio, and cognitive impairment was found in the total population, the hypertensive subgroup, or the non-hypertensive subgroup.

Regarding the relationship between plasma Aβ levels and cognitive function, previous population-based studies have shown great heterogeneity in results. A relatively large number of studies suggest that lower plasma Aβ_42_ levels or plasma Aβ_42_/Aβ_40_ ratios may be associated with an increased risk of AD or poorer cognitive function (Brickman et al., 2021; de Wolf et al., 2020; Giudici et al., 2020; Rembach et al., 2014). However, a few studies report the opposite, indicating that higher plasma Aβ_42_ levels or plasma Aβ_42_/Aβ_40_ ratios are associated with higher AD risk or poorer cognitive function (Chen et al., 2019; Cullen et al., 2021). Additionally, some researches support an association between plasma Aβ_40_ levels and the risk of AD or dementia (Botella Lucena et al., 2022; Hansson et al., 2012; Sullivan et al., 2021; van Oijen et al., 2006). Conversely, other studies have found no significant relationship between plasma Aβ levels and AD or cognitive function (Donohue et al., 2015; Hsu et al., 2017). The reasons for this heterogeneity may be multifaceted. First, recent studies suggest a two-stage phenomenon for blood Aβ_42_ in AD pathogenesis: plasma Aβ_42_ levels increase in the pre-pathological stage and then decrease as Aβ accumulates in the brain (Botella Lucena et al., 2022), a pattern supported by another study as well (Schupf et al., 2008). Therefore, blood samples taken at different disease stages may vary significantly. Second, plasma Aβ levels may be influenced by factors such as age (Sullivan et al., 2021) and blood pressure (Abdullah et al., 2009; Lambert et al., 2011). Furthermore, plasma Aβ has multiple sources: it can be transported from Aβ in the brain (Tarasoff-Conway et al., 2015), or generated from peripheral sources such as platelets (Carbone et al., 2021), so it is also affected by peripheral clearance capabilities (e.g., liver and kidney function; Tarasoff-Conway et al., 2015). Overall, cerebrospinal fluid Aβ_42_ or the Aβ_42_/Aβ_40_ ratio are considered reliable biomarkers for AD diagnosis or preclinical prediction, while plasma Aβ levels have yet to demonstrate consistent effectiveness for diagnosing or predicting AD or cognitive function.

This study contributes new insights into the relationship between plasma Aβ and cognitive impairment, identifying hypertension as a key moderator in this association. Among hypertensive individuals, simultaneous increases in plasma Aβ_40_ and Aβ_42_ levels were associated with cognitive impairment risk, rather than isolated changes in Aβ_40_, Aβ_42_, or their ratio. To our knowledge, the simultaneous elevation of plasma Aβ_40_ and Aβ_42_ as a predictor of cognitive impairment has not been explored in previous studies. Additionally, lower plasma Aβ_40_ was significantly associated with an increased risk of cognitive impairment in non-hypertensive individuals, but not in those with hypertension. These findings suggest that hypertension may exacerbate the dysregulation of Aβ production or clearance, potentially increasing the risk of cognitive impairment. The underlying mechanisms likely involve hypertension-induced pathways such as aggravated neurovascular dysfunction (Faraco et al., 2016) and impaired glymphatic clearance (Mortensen et al., 2019), which compromise the brain’s ability to manage Aβ levels effectively. Understanding these processes could inform targeted interventions aimed at mitigating cognitive impairment risk in hypertensive populations.

A large number of studies have confirmed hypertension as a risk factor for AD (Livingston et al., 2020; Scheltens et al., 2021) and some has demonstrated its role in modulating the relationship between brain Aβ accumulation and cognitive decline. Specifically, one study shows that cognitive decline occurs more rapidly in hypertensive patients when there are equivalent levels of Aβ deposition in the brain (Clark et al., 2019). Another reports that Low SBP untreated by antihypertensive medications is associated with significantly decreased risk of dementia and less cerebral Aβ (Kuller et al., 2022). Further, some studies suggest that blood pressure may also influence plasma Aβ levels (Abdullah et al., 2009; Lambert et al., 2011), either directly (She et al., 2021) or in combination with other vascular factors (Sapkota et al., 2023). These support the biological plausibility that hypertension could influence the association between plasma Aβ levels and cognitive impairment. The present study provides additional evidence for this hypothesis. In future similar studies, hypertension should be considered as a moderator rather than a confounder, to help clarify findings and reduce variability in research outcomes.

Despite the authors’ best efforts, this study has some shortcomings. As a cross-sectional study, it establishes associations between plasma Aβ levels and cognitive impairment but does not allow for causal inference, limiting the ability to predict longitudinal outcomes. Secondly, the diagnosis of cognitive impairment in this study was solely based on the MMSE score. While the MMSE is widely used in many high-quality population-based studies, it is important to acknowledge its’ limitations, including reduced sensitivity for detecting mild cognitive impairment (MCI) and early stages of cognitive decline. Additionally, its specificity can also be influenced by factors such as education level, cultural background, and socioeconomic status. This is particularly relevant to our study population of rural residents in Xi’an, China, where lower education levels are common, and cognitive tasks on the MMSE may lack cultural relevance or fail to align with the practical, experience-based cognitive strengths typical of rural populations. Although we used different cutoff value to adjust for different education levels, there remains a possibility of false positives and false negatives in this study. In addition, this study was unable to distinguish between cognitive impairment caused by AD and cognitive impairment caused by vascular dementia (VD). However, considering that hypertension can affect cognitive function through both the AD pathway and the VD pathway (Tzourio et al., 2014), therefore, in studies on hypertension, cognitive function and its biomarkers, it is relatively reasonable to use all-cause cognitive impairment as the primary outcome. Finally, as an exploratory study, the hypertension-specific association between plasma Aβ and cognitive impairment found in this study requires further empirical research to confirm these findings and assess its potential as a diagnostic indicator for cognitive impairment.

## 5 Conclusion

The present study showed that simultaneously elevated plasma Aβ_40_ and Aβ_42_ in individuals with hypertension, and decreased plasma Aβ_40_ in those without hypertension, served as risk factors for cognitive impairment. These findings indicate that hypertension is an important modulator in the relationship between plasma Aβ and cognitive impairment. To enhance predictive accuracy, future research should account for hypertension status when investigating plasma Aβ levels as potential indicators of cognitive impairment risk. What’s more, these findings suggest that incorporating hypertension status into cognitive screening protocols could improve the identification of individuals at higher risk for cognitive impairment. Furthermore, the potential role of hypertension in exacerbating Aβ dysregulation highlights the need for hypertension-specific interventions to mitigate cognitive impairment risk.

## Data Availability

The raw data supporting the conclusions of this article will be made available by the authors, without undue reservation.
