# Supplementary material for: Hypertension moderates the relationship between plasma beta-amyloid and cognitive impairment: a cross-sectional study in Xi’an, China
Source: Front Aging Neurosci. 2025 Jan 28;17:1532676. doi: 10.3389/fnagi.2025.1532676 (PMC11810958; doi:10.3389/fnagi.2025.1532676)
Supplement: Supplementary file 1 [file Data_Sheet_1.docx]

Supplementary Material

# Supplementary Tables

**Stable 1. Risk of** **cognitive impairment according to categories-2 of combined plasma Aβ in total population, hypertension subgroup and non-hypertension subgroup.**

| **Variables** | **Β** | **S.E.** | **Wald** | **OR** | **95% CI** | ***P*** |
| --- | --- | --- | --- | --- | --- | --- |
| **Categories-2 of combined plasma Aβ in total** |  |  |  |  |  |  |
| **L-Aβ_40_ and L- Aβ_42_ (reference)** | 0 | - | - | 1 | - | - |
| **H-Aβ_40_ and L- Aβ_42_** | 0.030 | 0.216 | 0.019 | 1.030 | 0.675-1.573 | 0.890 |
| **L-Aβ_40_ and H- Aβ_42_** | 0.254 | 0.209 | 1.475 | 1.289 | 0.856-1.943 | 0.225 |
| **H-Aβ_40_ and H- Aβ_42_** | 0.369 | 0.312 | 1.397 | 1.446 | 0.785-2.663 | 0.237 |
| **Categories-2 of combined plasma Aβ in HP** |  |  |  |  |  |  |
| **L-Aβ_40_ and L- Aβ_42_ (reference)** | 0 | - | - | 1 | - | - |
| **H-Aβ_40_ and L- Aβ_42_** | 0.127 | 0.276 | 0.210 | 1.135 | 0.661-1.950 | 0.647 |
| **L-Aβ_40_ and H- Aβ_42_** | 0.230 | 0.292 | 0.621 | 1.259 | 0.710-2.233 | 0.431 |
| **H-Aβ_40_ and H- Aβ_42_** | 0.903 | 0.391 | 5.340 | 2.466 | 1.447-5.303 | 0.021 |
| **Categories-2 of combined plasma Aβ in non-HP** |  |  |  |  |  |  |
| **L-Aβ_40_ and L- Aβ_42_ (reference)** | 0 | - | - | 1 | - | - |
| **H-Aβ_40_ and L- Aβ_42_** | -0.073 | 0.356 | 0.042 | 0.930 | 0.463-1.868 | 0.838 |
| **L-Aβ_40_ and H- Aβ_42_** | 0.289 | 0.310 | 0.873 | 1.336 | 0.728-2.451 | 0.350 |
| **H-Aβ_40_ and H- Aβ_42_** | -0.450 | 0.588 | 0.586 | 0.638 | 0.201-2.018 | 0.444 |

Multivariate logistic regression models were established with cognitive impairment (yes or no) as the dependent variable and categories-2 of combined plasma Aβ as the independent variable in total population, the hypertension subgroup and the non-hypertension subgroup. The adjusted confounders included age, sex, years of education, smoking, drinking, lack of physical activity, heart disease, stroke, mean arterial pressure, BMI, FBG, TG, TC, LDL and HDL.

Categories-2 of combined plasma Aβ were created by making bisection of plasma Aβ_40_ (L-Aβ_40_<58 pg/ml and H-Aβ_40_≥58 pg/ml) and plasma Aβ_42_ (L-Aβ_42_<45 pg/ml and H-Aβ_42_≥45 pg/ml) according 75th percentile, and combining them as L-Aβ_40_ and L-Aβ_42_, H-Aβ_40_ and L-Aβ_42_, L-Aβ_40_ and H-Aβ_42_, H-Aβ_40_ and H-Aβ_42_.

**Stable 2. Risk of cognitive impairment for subjects with no stroke history at different levels of plasma Aβ.**

| **Variables** | | **Β** | **S.E.** | | **Wald** | | **OR** | | **95% CI** | | ***P*** | | |
| --- | --- | --- | --- | --- | --- | --- | --- | --- | --- | --- | --- | --- | --- |
| **Categories of combined plasma Aβ in total without stroke** | |  |  | |  | |  | |  | |  | | |
| **L-Aβ_40_ and L- Aβ_42_ (reference)** | | 0 | - | | - | | 1 | | - | | - | | |
| **H-Aβ_40_ and L- Aβ_42_** | | -0.164 | 0.258 | | 0.404 | | 0.849 | | 0.512-1.407 | | 0.525 | | |
| **L-Aβ_40_ and H- Aβ_42_** | | -0.35 | 0.3 | | 1.365 | | 0.704 | | 0.391-1.268 | | 0.243 | | |
| **H-Aβ_40_ and H- Aβ_42_** | | 0.221 | 0.249 | | 0.785 | | 1.247 | | 0.765-2.033 | | 0.376 | | |
| **Categories of combined plasma Aβ in HP without stroke** | |  |  | |  | |  | |  | |  | | |
| **L-Aβ_40_ and L- Aβ_42_ (reference)** | | 0 | - | | - | | 1 | | - | | - | | |
| **H-Aβ_40_ and L- Aβ_42_** | | 0.341 | 0.402 | | 0.72 | | 1.407 | | 0.640-3.093 | | 0.396 | | |
| **L-Aβ_40_ and H- Aβ_42_** | | -0.072 | 0.458 | | 0.025 | | 0.93 | | 0.379-2.283 | | 0.875 | | |
| **H-Aβ_40_ and H- Aβ_42_** | | 0.88 | 0.389 | | 5.121 | | 2.41 | | 1.125-5.163 | | 0.024 | | |
| **Categories of combined plasma Aβ in non-HP without stroke** | |  |  | |  | |  | |  | |  | | |
| **L-Aβ_40_ and L- Aβ_42_ (reference)** | | 0 | - | | - | | 1 | | - | | - | | |
| **H-Aβ_40_ and L- Aβ_42_** | | -0.567 | 0.359 | | 2.487 | | 0.567 | | 0.281-1.148 | | 0.115 | | |
| **L-Aβ_40_ and H- Aβ_42_** | | -0.519 | 0.412 | | 1.588 | | 0.595 | | 0.266-1.334 | | 0.208 | | |
| **H-Aβ_40_ and H- Aβ_42_** | | -0.32 | 0.356 | | 0.806 | | 0.726 | | 0.361-1.460 | | 0.369 | | |
| **Decreased Aβ_40_ in total without stroke** | | 0.293 | | | 0.231 | | 1.615 | | 1.341 | | 0.853-2.107 | | 0.204 |
| **Decreased Aβ_40_ in HP without stroke** | | 1.058 | | | 0.404 | | 6.852 | | 0.347 | | 0.157-0.767 | | 0.009 |
| **Decreased Aβ_40_ in non-HP without stroke** | | -0.231 | | | 0.302 | | 0.588 | | 1.260 | | 0.698-2.276 | | 0.443 |

Multivariate logistic regression models were established with cognitive impairment (yes or no) as the dependent variable and categories of combined plasma Aβ as the independent variable in total population without stroke history, the hypertension without stroke history subgroup and the non-hypertension without stroke history subgroup. The adjusted confounders included age, sex, years of education, smoking, drinking, lack of physical activity, heart disease, mean arterial pressure, BMI, FBG, TG, TC, LDL and HDL.

Categories of combined plasma Aβ were created by making bisection of plasma Aβ_40_ (L-Aβ_40_<52 pg/ml and H-Aβ_40_≥52 pg/ml) and plasma Aβ_42_ (L-Aβ_42_<41 pg/ml and H-Aβ_42_≥41 pg/ml) according to average value, and combining them as L-Aβ_40_ and L-Aβ_42_, H-Aβ_40_ and L-Aβ_42_, L-Aβ_40_ and H-Aβ_42_, H-Aβ_40_ and H-Aβ_42_. Decreased plasma Aβ_40_ was defined as plasma Aβ_40_ lower than 25th percentile (<46pg/ml).

**Stable 3. Risk of cognitive impairment between treated-HP and non-treated-HP groups at different levels of plasma Aβ.**

| **Variables** | **Β** | **S.E.** | **Wald** | **OR** | **95% CI** | ***P*** |
| --- | --- | --- | --- | --- | --- | --- |
| **Categories of combined plasma Aβ in treated-HP** |  |  |  |  |  |  |
| **L-Aβ_40_ and L- Aβ_42_ (reference)** | 0 | - | - | 1 | - | - |
| **H-Aβ_40_ and L- Aβ_42_** | 1.112 | 0.755 | 2.17 | 3.041 | 0.692-13.36 | 0.141 |
| **L-Aβ_40_ and H- Aβ_42_** | 0.196 | 0.836 | 0.055 | 1.217 | 0.236-6.264 | 0.814 |
| **H-Aβ_40_ and H- Aβ_42_** | 0.726 | 0.816 | 0.792 | 2.067 | 0.417-10.237 | 0.374 |
| **Categories of combined plasma Aβ in non-treated-HP** |  |  |  |  |  |  |
| **L-Aβ_40_ and L- Aβ_42_ (reference)** | 0 | - | - | 1 | - | - |
| **H-Aβ_40_ and L- Aβ_42_** | -0.273 | 0.469 | 0.339 | 0.761 | 0.304-1.908 | 0.561 |
| **L-Aβ_40_ and H- Aβ_42_** | -0.593 | 0.549 | 1.165 | 0.553 | 0.188-1.622 | 0.28 |
| **H-Aβ_40_ and H- Aβ_42_** | 0.836 | 0.431 | 3.756 | 2.307 | 0.991-5.374 | 0.053 |

Multivariate logistic regression models were established with cognitive impairment (yes or no) as the dependent variable and categories of combined plasma Aβ as the independent variable in treated-HP group and the non-treated-HP group. The adjusted confounders included age, sex, years of education, smoking, drinking, lack of physical activity, heart disease, stroke, mean arterial pressure, BMI, FBG, TG, TC, LDL and HDL.

Categories of combined plasma Aβ were created by making bisection of plasma Aβ_40_ (L-Aβ_40_<52 pg/ml and H-Aβ_40_≥52 pg/ml) and plasma Aβ_42_ (L-Aβ_42_<41 pg/ml and H-Aβ_42_≥41 pg/ml) according to average value, and combining them as L-Aβ_40_ and L-Aβ_42_, H-Aβ_40_ and L-Aβ_42_, L-Aβ_40_ and H-Aβ_42_, H-Aβ_40_ and H-Aβ_42_.

# Supplementary Figure


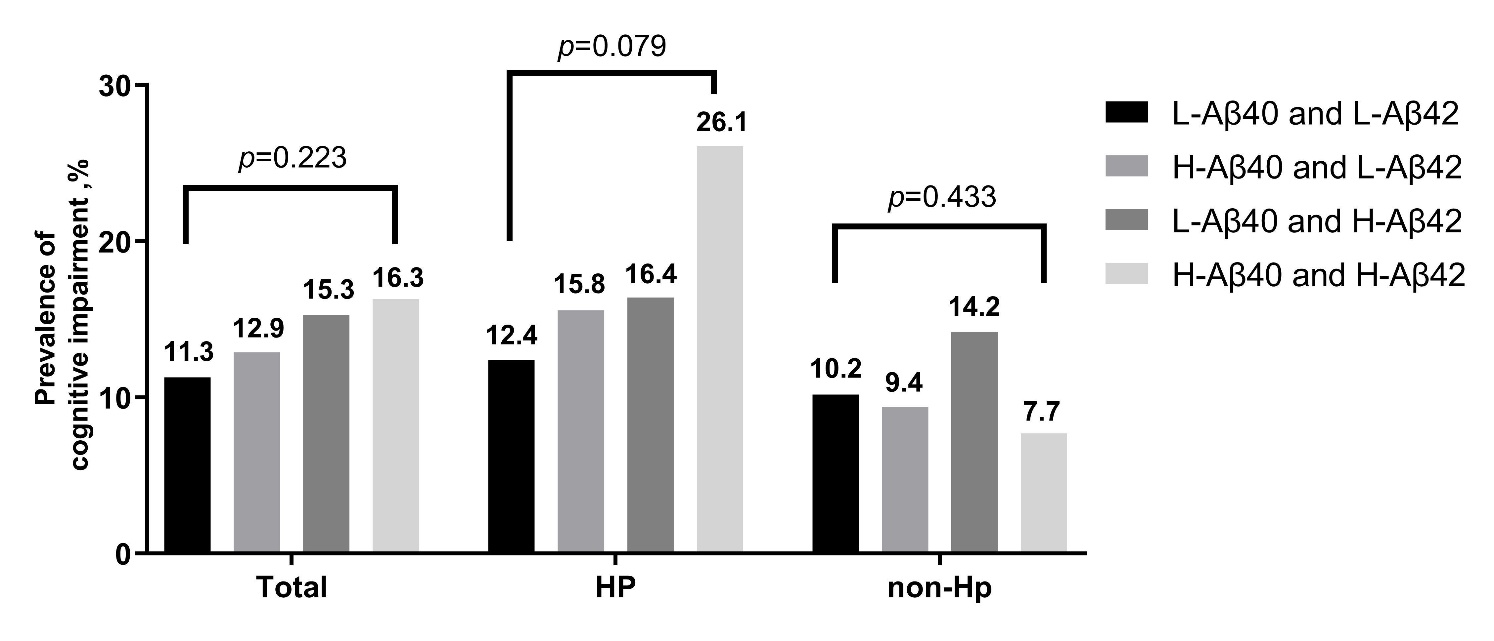


**Sfigure 1.** The prevalence of cognitive impairment according to categories-2 of combined plasma Aβ. Categories-2 of combined plasma Aβ were created by making bisection of plasma Aβ_40_ (L-Aβ_40_<58 pg/ml and H-Aβ_40_≥58 pg/ml) and plasma Aβ_42_ (L-Aβ_42_<45 pg/ml and H-Aβ_42_≥45 pg/ml) according to 75th percentile, and combining them as L-Aβ_40_ and L-Aβ_42_, H-Aβ_40_ and L-Aβ_42_, L-Aβ_40_ and H-Aβ_42_, H-Aβ_40_ and H-Aβ_42_.
